# Supplementary material for: Hybrid Models and Biological Model Reduction with PyDSTool
Source: PLoS Comput Biol. 2012 Aug 9;8(8):e1002628. doi: 10.1371/journal.pcbi.1002628 (PMC3415397; doi:10.1371/journal.pcbi.1002628)
Supplement: Text S4 — Complete source code for the PyDSTool package (version 0.88.120504). Includes API documentation and help files linking to web pages. This file is identical to the current public release on Sourceforge.net. (ZIP) [file pcbi.1002628.s004.zip › PyDSTool/html/PyDSTool.Toolbox.optimizers.tests.test_powell-pysrc.html]

xml version="1.0" encoding="ascii"?


PyDSTool.Toolbox.optimizers.tests.test\_powell


| Home | Trees | Indices | Help | PyDSTool |
| --- | --- | --- | --- | --- |

Package PyDSTool ::
Package Toolbox ::
Package optimizers ::
Package tests ::
Module test\_powell

[hide private]

[frames] | no frames]

# Source Code for Module PyDSTool.Toolbox.optimizers.tests.test\_powell

```
 1  #!/usr/bin/env python
 
 2  
 
 3  # Matthieu Brucher
 
 4  # Last Change : 2007-10-16 20:48
 
 5  
 
 6  """
 
 7  Class defining the Powell function
 
 8  """ 
 9  
 
10  import numpy 
11  from numpy.testing import * 
12  set_package_path() 
13  from optimizers import criterion, step, optimizer, line_search 
14  restore_path() 
15  
 
16  
 


17 -class Powell:


18    """
 
19    The Powell function
 
20    """ 


21 -  def __call__(self, x):


22      """
 
23      Get the value of the Powell function at a specific point
 
24      """ 
25      return (x[0] + 10* x[1])**2 + 5 * (x[2] - x[3])**2 + (x[1] - 2 * x[2])**4 + 10 * (x[0] - x[3])**4

26  
 


27 -  def gradient(self, x):


28      """
 
29      Evaluates the gradient of the function
 
30      """ 
31      return numpy.array([2 * (x[0] + 10 * x[1]) + 40 * (x[0] - x[3])**3, 20 * (x[0] + 10 * x[1]) + 4 * (x[1] - x[2])**3, 10 * (x[2] - x[3]) - 8 * (x[1] - 2 * x[2])**3, 10 * (x[2] - x[3]) - 40 * (x[0] - x[3])**3], dtype = numpy.float)

32  
 


33 -  def hessian(self, x):


34      """
 
35      Evaluates the gradient of the function
 
36      """ 
37      print x, self(x) 
38      return numpy.array([[2 + 120 * (x[0] - x[3]) ** 2, 20, 0, -120 * (x[0] - x[3]) ** 2], [20, 200 + 12 * (x[1] - 2 * x[2]) ** 2, -24 * (x[1] - 2 * x[2]) ** 2, 0], [0, -24 * (x[1] - 2 * x[2]) ** 2, 10 + 48 * (x[1] - 2 * x[2]) ** 2, -10], [-120 * (x[0] - x[3]) ** 2, 0, -10, -10 + 120 * (x[0] - x[3])**2]], dtype = numpy.float)

39  
 


40 -class test_Powell(NumpyTestCase):


41    """
 
42    Global test class with the Powell function
 
43    """ 


44 -  def check_simple_newton(self):


45      startPoint = numpy.empty(4, numpy.float) 
46      startPoint[0] = 3. 
47      startPoint[1] = -1. 
48      startPoint[2] = 0. 
49      startPoint[3] = 1. 
50      optimi = optimizer.StandardOptimizer(function = Powell(), step = step.NewtonStep(), criterion = criterion.OrComposition(criterion.RelativeValueCriterion(0.00000001), criterion.IterationCriterion(1000)), x0 = startPoint, line_search = line_search.SimpleLineSearch()) 
51      assert_array_almost_equal(optimi.optimize(), numpy.zeros(4, numpy.float), decimal=2)

52  
 
53  if __name__ == "__main__": 
54      NumpyTest().run() 
55
```

  


| Home | Trees | Indices | Help | PyDSTool |
| --- | --- | --- | --- | --- |

|  |  |
| --- | --- |
| Generated by Epydoc 3.0.1 on Fri May 4 15:24:16 2012 | http://epydoc.sourceforge.net |
